# Soy Isoflavones Genistein and Daidzein Exert Anti-Apoptotic Actions via a Selective ER-mediated Mechanism in Neurons following HIV-1 Tat_1–86_ Exposure

**DOI:** 10.1371/journal.pone.0037540

**Published:** 2012-05-22

**Authors:** Sheila M. Adams, Marina V. Aksenova, Michael Y. Aksenov, Charles F. Mactutus, Rosemarie M. Booze

**Affiliations:** Department of Psychology, University of South Carolina, Columbia, South Carolina, United States of America; Virgen Macarena University Hospital, Spain

## Abstract

**Background:**

HIV-1 viral protein Tat partially mediates the neural dysfunction and neuronal cell death associated with HIV-1 induced neurodegeneration and neurocognitive disorders. Soy isoflavones provide protection against various neurotoxic insults to maintain neuronal function and thus help preserve neurocognitive capacity.

**Methodology/Principal Findings:**

We demonstrate in primary cortical cell cultures that 17β-estradiol or isoflavones (genistein or daidzein) attenuate Tat_1–86_-induced expression of apoptotic proteins and subsequent cell death. Exposure of cultured neurons to the estrogen receptor antagonist ICI 182,780 abolished the anti-apoptotic actions of isoflavones. Use of ERα or ERβ specific antagonists determined the involvement of both ER isoforms in genistein and daidzein inhibition of caspase activity; ERβ selectively mediated downregulation of mitochondrial pro-apoptotic protein Bax. The findings suggest soy isoflavones effectively diminished HIV-1 Tat-induced apoptotic signaling.

**Conclusions/Significance:**

Collectively, our results suggest that soy isoflavones represent an adjunctive therapeutic option with combination anti-retroviral therapy (cART) to preserve neuronal functioning and sustain neurocognitive abilities of HIV-1 infected persons.

## Introduction

HIV-1 infection of the central nervous system (CNS) causes several neurological disorders, known as HIV-associated neurocognitive disorders (HAND) [1]. Although the incidence of severe dementia has significantly decreased since the advent of combination anti-retroviral therapy (cART), cognitive and motor impairments persist in up to 50% of HIV-1 positive patients due to longer life expectancy, the lack of patient compliance with cART therapy and the low penetrability of cART into the CNS [2]. The continued prevalence of neurological dysfunction suggests cART fails to provide complete protection from the development of HAND [1], [3], [4] and there currently are no pharmacotherapies targeted to HAND.

HIV-1 enters the brain early after infection and, although, HIV-1 does not infect neurons, neuronal dysfunction is key in HIV pathogenesis [5]–[7]. The early viral proteins such as Tat are continually produced despite the presence of cART [7]–[10]. Accordingly, Tat is rapidly taken up by neuronal cells and has been shown to have direct toxic effects on neurons though various mechanisms. Studies have shown Tat to mediate excitotoxicity via NMDA receptors [11]–[13], synaptic damage and dendritic pruning [14], induce apoptotic cascades [15], [16], calcium dysregulation [17], oxidative stress [18], and dopaminergic system dysfunction [19], [20]. Tat exposure has been shown to negatively affect cognitive processes in animal models [21], [22]. The observations that the viral regulatory protein Tat is actively secreted by infected cells, and that Tat mRNA is elevated in patients with HIV-1 suggest a possible role of extracellular Tat in the progression of HIV-1-induced neurodegeneration [23]–[25].

Phytoestrogens, such as the soy isoflavones genistein and daidzein, mimic the neuroprotective actions and functions of estrogen in the brain, as they bind to the estrogen receptor (ER) and affect estrogen-mediated processes [26]–[29]. Several studies have found that soy isoflavones can improve cognitive functions in both humans and rats, but underlying mechanisms remain unknown [30]–[35]. Additional studies have shown that isoflavones have neuroprotective effects against various neurodegenerative insults. Genistein and daidzein have demonstrated neuroprotective efficacy against glutamate excitotoxicity and Aβ_25–35_ induced loss of cell viability, oxidative stress and initiation of apoptosis in hippocampal neurons [36], [37]. As the dopaminergic system is sensitive to HIV in the brain, isoflavones, similar to estradiol, may interact with dopamine to preserve motor and cognitive functions [35], [38]–[40].

Estrogen therapy is met with resistance due to its association with reproductive and breast cancers [41], [42]. However, dietary consumption and supplementation with soy isoflavones is widespread. Consuming a typical Western diet yields low nanomolar concentrations of circulating isoflavones [43], [44]. In people consuming modest amount of soy products yielding ∼50 mg/day of total isoflavones, plasma levels of 50–800 ng/ml have been achieved for daidzein and genistein [43], which is comparable to concentrations observed in a traditional Japanese diet [44]. However, consuming a western vegetarian diet and taking supplements has been shown to achieve increased isoflavone consumption comparable to or higher than Asian levels [44]–[46]. Furthermore, human studies that have reported improved cognitive function with soy isoflavone consumption have used ∼60–100 mg/day doses of isoflavones [31], [34]. The cognitive improvements seen with high doses of soy in vivo were not associated with abnormalities in reproductive health of humans, including men [31], [47]–[49]. This broadens the use and benefits of these estrogenic compounds to not only women but also men.

Soy isoflavones preferentially binding to ERβ is of significant consideration in neuroprotection as this ER subtype is highly expressed in the brain compared to ERα, which due to its high expression in the reproductive tissue, has been associated with the proliferative effects of estrogen. Elucidating whether isoflavone protection is mediated by ER selectivity is a central focus in developing neuroprotective strategies. In the current experiments, we investigated whether treatment with soy isoflavones, genistein or daidzein, could attenuate HIV-1 Tat-induced mitochondria associated apoptosis in cortical cell cultures**.** Further, we explored whether isoflavone neuroprotection against HIV-1 viral protein Tat-induced neural toxicity involves ER-mediated attenuation of apoptotic signaling. We demonstrated that isoflavones maintained neuronal cell viability in the presence of prolonged Tat exposure. We also observed that isoflavones prevented Tat-induced upregulation of mitochondrial apoptotic cascade regulators. Moreover, we determined that the protective actions of isoflavones were mediated by estrogen receptors.

## Results

### Physiological Doses of Genistein and Daidzein Prevent Cell Death Following Tat_1–86_ Exposure

**Figure 1:**
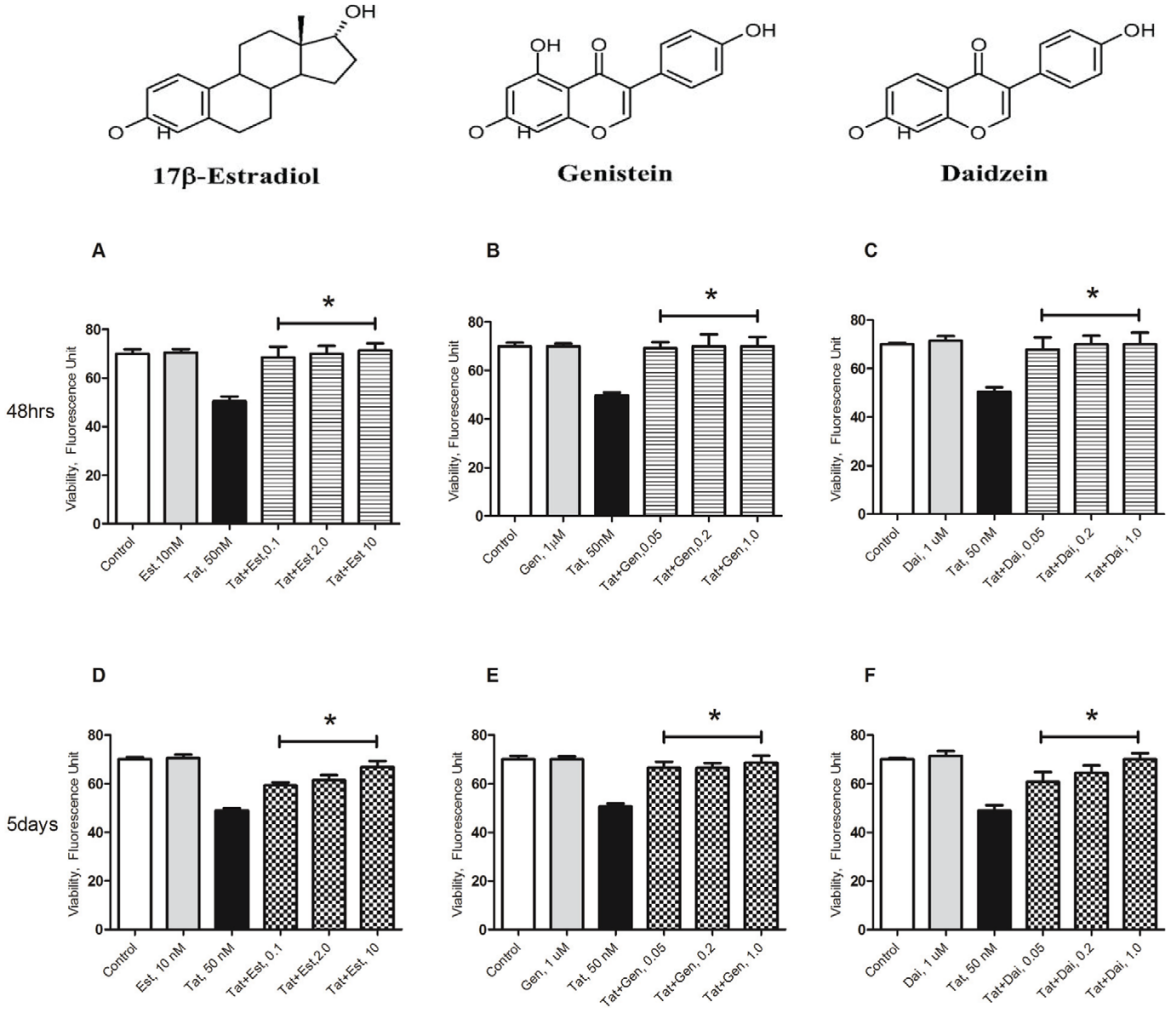
Soy isoflavones genistein and daidzein protect primary cortical cultures from Tat neurotoxicity. Primary cortical neurons were exposed to estrogen (0.1, 2.0 and 10 nM), or isoflavones (0.05, 0.2 and 1 µM) 24 hr prior to the start of Tat_1–86_ B (50 nM) treatment. Cell viability was assessed by Live/Dead assay. Live/Dead ratios were determined after 48 hr (**A-C**) or 5 days (**D-F**) of the continuous exposure to Tat or equal volume of vehicle in cell culture groups that were treated or not treated with estrogen, GEN, or DAI. Data represents mean values ± SEM, n of cultures analyzed  = 7–12 per each group. *- indicates significant (*p*≤0.05) protective effects of the selected compounds against Tat neurotoxicity (cell viability decrease) in cortical cell cultures. Repeated (2–3) trials using cell culture preparations from different litters were carried out to ensure the reproducibility of the results.

We have previously shown that 17β-estradiol attenuated Tat-induced cell death [50]. As shown in Figure 1, the cell viability decrease (≈25% of control) induced by prolonged (up to 5 days) exposure to the toxic dose of Tat_1–86_ B was abrogated by 0.1–10 nM of 17β-estradiol. Similar alleviation of Tat-induced neuronal cell death was observed when isoflavones GEN or DAI were used at doses 0.05, 0.2, and 1 µM (Figure 1). Our levels of 50 nM to 1.0 µM of genistein and daidzein are within the range of observed plasma levels of isoflavones following consumption of soy products which reflect ∼200 nM-3 µM. Results indicate that physiologically relevant concentrations of isoflavones are able to effectively protect cortical neurons against Tat toxicity *in vitro*.

### Genistein and Daidzein Attenuate Tat-induced Caspase Activation in Primary Cortical Cell Cultures

**Figure 2:**
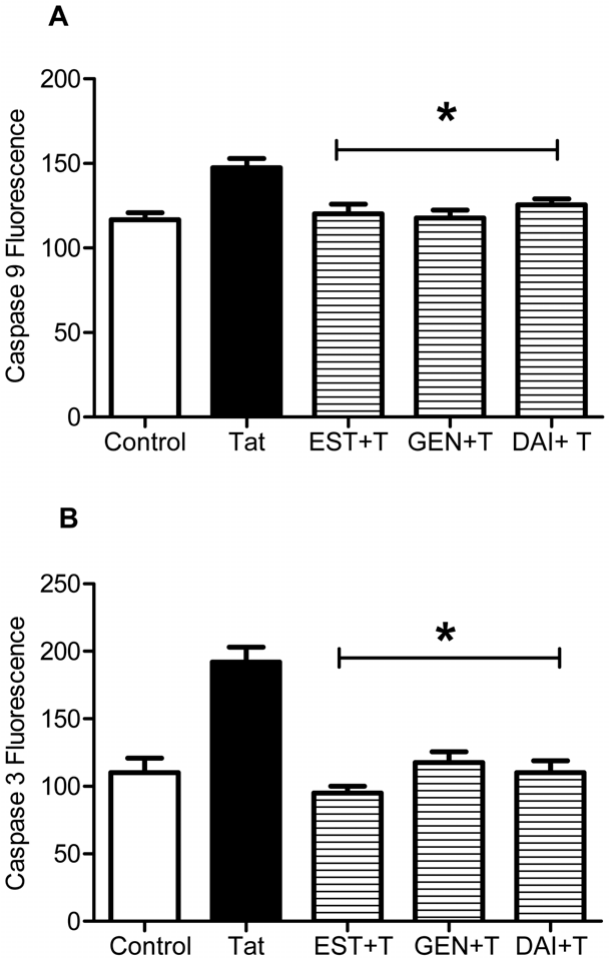
Genistein and daidzein attenuate Tat-induced caspase activation in primary cortical cultures. Cortical cultures were treated with 1 µM GEN or DAI 24 hr prior to Tat exposure. Expression of activated apoptotic proteins **A**. Caspase 9 (4 hr of Tat exposure) and **B**. caspase 3 (4 hr Tat exposure) was assessed by ELISA experiments. Data represents mean values ± SEM, with experiments performed in triplicate, *p≤0.05 as compared to Tat-treated cultures.

In the present experiments, we evaluated if the protective potential of GEN and DAI against Tat involves downregulation of caspase activity. Figure 2A shows significant caspase 9 activation following only 4 hr of Tat exposure (*p≤*0.05). Preincubation with 10 nM 17β-estradiol or 1 µM of isoflavones (GEN or DAI) prevented the increase in Tat-induced caspase 9 activation. Cultures pretreated with GEN or DAI displayed caspase 9 activity similar to that of estradiol treated cultures (*p*≤0.05). Moreover, analysis revealed that caspase 9 activity was not statistically different between the 17β-estradiol, GEN and DAI pretreated cultures.

In regard to effector caspase 3 activity, a similar effect was observed with phytoestrogen pretreatment prior to Tat exposure. These results demonstrated a significant increase in caspase 3 activation in cortical cultures following 4 hr exposure to Tat (*p*≤0.05). The current experiments show that the addition of GEN or DAI prior to incubation with 50 nM Tat significantly attenuated the upregulation of active caspase 3 expression (*p≤*0.05, Figure 2B). The level of activation of caspase 3 in GEN and DAI pretreatment groups was not significantly different from that of vehicle-treated controls; furthermore, these levels were very similar to that of the 17β-estradiol treated cultures. These results indicate that isoflavones GEN and DAI downregulate Tat-induced caspase activation to a level comparable to that of 17β-estradiol, suggesting that isoflavones and estradiol may share a common neuroprotective mechanism.

### Genistein and Daidzein Sustain Levels of Mitochondrial Proteins Bax and Bcl-2 Expression Following Tat Exposure

**Figure 3:**
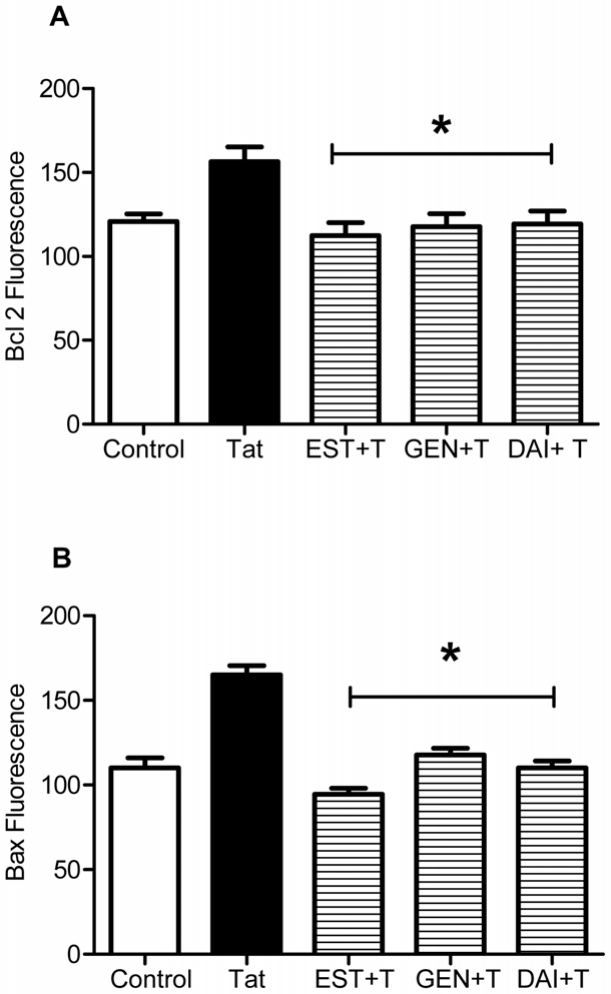
Isoflavones prevent Tat-induced expression of Bcl-2 and Bax protein levels. Cortical cultures were treated with 1 µM GEN or DAI 24 hr prior to Tat exposure. Expression of apoptotic proteins **A**. Bcl-2 (16 hr of Tat exposure) and **B**. Bax (4 hr Tat exposure) were assessed by ELISA experiments. Data represents mean values ± SEM, with experiments performed in triplicate, *p≤0.05 as compared to Tat-treated cultures.

The protection of cortical cell cultures with 17β-estradiol against Tat toxicity is associated with regulating the effects of apoptotic proteins linked to the mitochondrial apoptotic pathway [50]. Therefore, we compared effects of neuroprotective concentrations of 17β-estradiol and isoflavones that completely eliminate Tat-induced death of cortical cells on the alterations in Bcl-2 and Bax protein levels. Results of the Bcl-2 ELISA presented in Figure 3A demonstrate that, similar to estradiol (10 nM), neuroprotective doses of GEN and DAI (1 µM) added to the cell culture medium 24 hr in advance of 50 nM Tat significantly (*p≤*0.05) attenuated the increase of Bcl-2 expression; an effect shown to occur within the first 16–24 hr of Tat exposure in cortical cell cultures [50].

We also evaluated the efficacy of isoflavones against Tat-induced Bax expression. Results of the Bax ELISA (Figure 3B) demonstrate that pretreatment with 1 µM GEN or DAI, or pretreatment with 10 nM estrogen, significantly (*p≤*0.05) blocks the induction of Bax expression in Tat-exposed cortical cells (Figure 3B). Effects of all the compounds on Tat-induced changes in Bcl-2/Bax protein expression were specific, since neither the exposure to 10 nM 17β-estradiol, nor the exposure to 1 µM isoflavones caused statistically significant changes in Bcl-2 or Bax immunoreactivities compared to non-treated control cell cultures.

### Estrogen Receptor Antagonists Block Anti-apoptotic Actions of Soy Isoflavones

**Figure 4:**
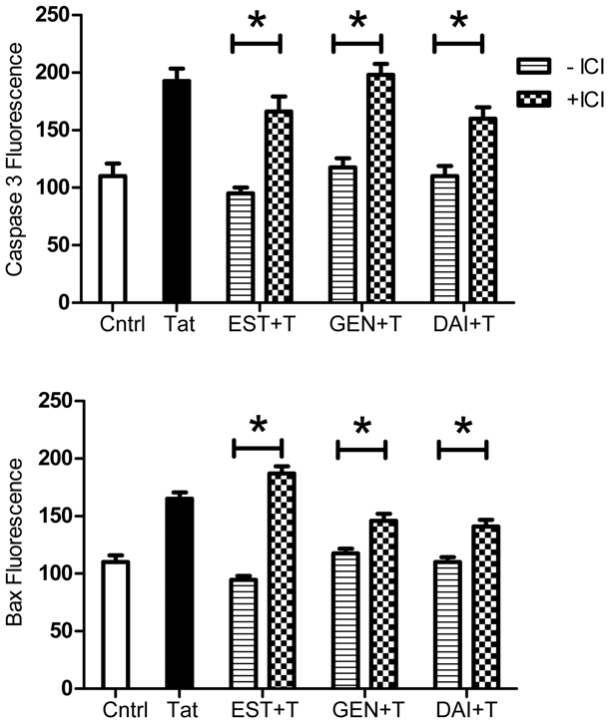
Estrogen receptors mediate isoflavone effects on Caspase 3 and Bax expression following Tat exposure. **A**. GEN or DAI effects on caspase 3 expression were blocked in the presence of ER antagonist, ICI 182,780. **B**. GEN or DAI effects on Tat-induced expression of Bax were reversed by ICI 182,780, suggesting that estrogenic actions on caspase 3 and Bax are ER mediated. Data represents mean values ± SEM, *p≤0.05 vs. GEN/DAI+T treated cultures.

Plant isoflavones, such as GEN and DAI, are similar to 17β-estradiol in chemical structure, which allows them to interact with estrogen receptors (ER). Neuroprotective effects of 17β-estradiol against Tat-induced apoptosis are mediated by two subtypes of estrogen receptors, ERα and ERβ. ER expression in primary cortical neurons used in the current experiments demonstrates the presence of both ER α and β immunoreactivity in our cultures (data not shown). In the present experiments, we sought to determine if the protective actions of isoflavones were ER-mediated. The ER antagonist, ICI 182,780 (100 nM), was added to cultures 1 hr prior to incubation with 17β-estradiol, GEN or DAI. Following 24 hr pretreatment, 50 nM Tat was added to cultures and its effects on the active caspase 3 (Figure 4A) and Bax (Figure 4B) protein levels have been analyzed.

As shown in Figure 4A, ICI prevented the downregulation of active caspase 3 by 17β-estradiol (10 nM), GEN or DAI (1 µM) pretreatment. Consistent with our previous study, ICI 182,780 averted estrogen-mediated activation of caspase 3 by Tat treatment of cortical cultures. Activated caspase 3 immunoreactivity in ICI+GEN+Tat-treated cultures was significantly higher than GEN+Tat treated cortical cultures (*p≤*0.05) and protein levels of activated caspase 3 were similar to that in Tat-only treated cultures. Similarly, the DAI effects on caspase 3 were sensitive to ICI inhibition of ERs. There was a significant increase in active caspase 3 expression with the addition of ICI compared to DAI+Tat treated cultures (*p*≤0.05). Results of the experiments indicate that GEN and DAI, similar to 17β-estradiol, inhibit the Tat-induced caspase 3 activation via an ER-mediated mechanism, as the addition of ICI 182,780 prior to estradiol returned caspase 3 activation levels to that of Tat only treated cultures. Moreover, caspase 3 activity did not differ statistically from that of Tat only treated cultures.

The results in Figure 4B show that GEN and DAI effects on Tat-induced Bax expression in cortical cultures are ICI-sensitive. A significant 25% increase in Bax expression was seen with ICI treatment prior to incubation with GEN or DAI (*p*≤0.05) (Figure 4B). Although, GEN and DAI possess estrogen-like activity, their affinity to ERα- or ERβ-subtypes is significantly different from 17β-estradiol. Therefore, we used specific antagonists of α or β ERs to evaluate selectivity of the effects of 17β-estradiol, GEN and DAI on Tat-induced changes in activated caspase 3 or Bax protein levels. The ER-subtype specific antagonists MPP (ERα) and PHTPP (ERβ) (1 µM) were added to cortical cultures prior to incubation with GEN or DAI and subsequent exposure to 50 nM Tat.

**Figure 5:**
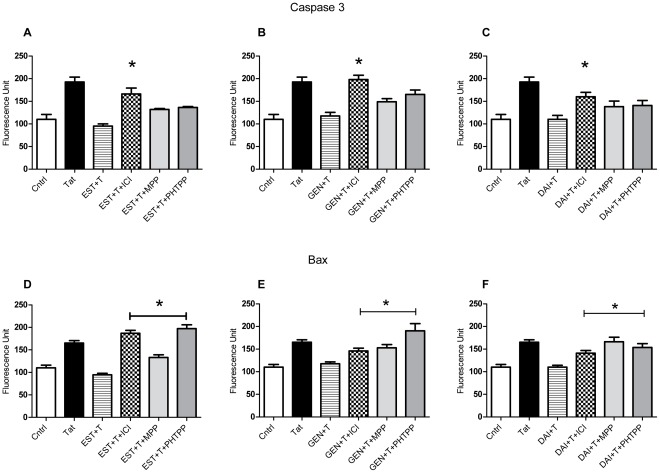
ER subtype specific effects against caspase activity and Bax expression. Similar to 17β-estradiol (**A**), GEN (**B**) and DAI (**C**) effects against Tat-induced caspase 3 activity were maintained in the presence of specific antagonists for ERα (MPP) and ERβ (PHTPP). ER subtype antagonists reveal that ERβ signaling was preferential for GEN (**E**) effects on Bax. DAI (**F**) effects on Bax were blocked in the presence of both ER subtype antagonists. Data represents mean values ± SEM, *p≤0.05 as compared to GEN/DAI+Tat treated cultures.

Neither α- nor β-selective ER antagonists were able to completely block the inhibitory effects of 10 nM 17β-estradiol (Figure 5A), or those of 1 µM GEN or DAI, on Tat-induced caspase 3 activation (Figure 5B, C). Although the ER subtype selective antagonists diminished the ability of all the compounds to decrease active caspase 3 protein levels in Tat-treated cultures, their effects were not statistically significant. Overall, selective antagonists of ERα- or ERβ-subtypes similarly affected the ability of both 17β-estradiol and isoflavones to downregulate Tat-induced caspase 3 activation.

The addition of ERα antagonist MPP did not significantly attenuate 17β-estradiol effects on Bax expression (Figure 5D), but was able to significantly decrease the effect of GEN and DAI. The ERβ-specific antagonist PHTPP completely blocked the effect of 17β-estradiol and GEN (Figure 5E, F). PHTPP also caused a partial, but significant, decrease in the ability of DAI to inhibit the Tat-dependent increase in Bax expression.

## Discussion

Neurocognitive deficits associated with HIV infection persist even with effective cART [51]. Targeting ERβ function may be a potential therapeutic option since it is highly expressed in the brain, specifically in cortical regions responsible for executive functions significantly affected in HIV-1 associated neurocognitive disorders (HAND) [35], [52]–[54]. In this study, we evaluated whether soy isoflavones, acting as ER selective compounds, were able to mimic the neuroprotective effects of estrogen in HIV-1 Tat_1–86_-exposed primary neuronal cultures. GEN and DAI represent soy isoflavones with ERα/β binding profiles showing much higher binding selectivity for ERβ than 17β-estradiol. Among soy phytoestrogens, GEN exhibits maximum ERα/β binding affinity with approximately 60-fold preference for ERβ over ERα. DAI has lower ERα/β binding affinity than GEN with 14-fold selectivity for ERβ binding. For comparison with these two soy isoflavones, 17β-estradiol’s preference for the ERβ binding is 0.78-fold [55]. GEN and DAI have very close chemical structures and are known to induce neuroprotective responses but at a much lower magnitude than 17β-estradiol.

Studies have demonstrated that oxidative stress and mitochondrial dysfunction coincide with Tat activation of apoptotic cascades [16], [38], [50], [56]. Moreover, we have recently reported that 17β-estradiol attenuated Tat-induced apoptotic signaling in an estrogen-receptor dependent manner [50]. We now demonstrate that the soy isoflavones genistein and daidzein prevent the upregulation of caspase activity in Tat-exposed cultures. In addition, upstream of caspase activation, we show that Tat exposure significantly increased expression of pro- and anti-apoptotic proteins Bax and Bcl-2 respectively, which regulate mitochondrial membrane permeability and thus, the release of apoptogenic substances. Our results indicate that treatment with GEN or DAI also markedly reduces the expression of Bax and Bcl-2 in Tat-exposed cortical cultures. A major finding of our study is that both soy isoflavones GEN and DAI exhibited protective effects similar to that exhibited by 17β-estradiol. GEN and DAI increased cell viability and attenuated the upregulation of apoptotic proteins in a manner comparable to that observed in estradiol treated cultures. At low micromolar doses we observed that isoflavones were able to maintain cell survival following prolonged exposure to Tat. These results suggested a similar neuroprotective action of isoflavones and 17β-estradiol involving inhibition of apoptotic pathways. Our experiments also demonstrated that selective ERβ agonists induce an anti-apoptotic effect in primary cultures exposed to HIV-1 Tat. Such observations support the findings that isoflavones are protective against oxidative stress-mediated apoptosis in HIV-1 infection.

Since isoflavones bind estrogen receptors (ERs), the neuroprotective actions of isoflavones may be produced through activation of the ER. We found that the addition of the ER antagonist ICI 182,780, which blocks both the ERα and ERβ subtypes, reversed GEN and DAI downregulation of caspase 3 activity and Bax expression with Tat exposure, suggesting that these effects of GEN and DAI were ER-dependent. The addition of ICI 182,780 had a more robust effect against genistein actions on caspase 3 activity, and sustained caspase levels similar to cultures treated with Tat. Furthermore, an isoflavone effect on Bax expression was also shown to be ER-dependent, as ICI 182,780 blocked genistein and daidzein inhibition of Tat-induced Bax expression. Collectively, our results suggest that genistein and daidzein act as estrogen receptor agonists in primary cortical neurons and activate estrogenic neural defense mechanisms.

Another major finding is that isoflavone anti-apoptotic effects are selective relative to estrogen receptor isoform. There are conflicting reports of which ER subtype, ERα or ERβ, specifically mediates the protective actions of estrogen [57], [58]. We observed in our previous studies with estradiol [50], inhibition of ERs with ICI 182,780 blocked the downregulation of apoptotic proteins in cultures pretreated with isoflavones. Further experiments sought to determine if these receptor-mediated effects were specific to a particular ER-subtype. Cultures in the presence of ERα-specific antagonist MPP or ERβ-specific antagonist PHTPP did not show specific attenuation of genistein and daidzein downregulation of caspase 3 activity. Similar to previous results observed with estradiol, both ER subtypes seem to play a role in genistein and daidzein inhibition of Tat activation of caspase 3. However, a more pronounced attenuation of genistein effects on Bax expression was observed in the presence of ERβ antagonist PHTPP. Thus, genistein effects on Bax expression may be preferential for ERβ-mediated signaling. Our results also demonstrated that the addition of both ER subtype antagonists significantly inhibited daidzein actions on Bax expression, suggesting involvement of both ERα and ERβ in daidzein downregulation of Bax. It is possible that daidzein’s lower binding affinity for ERs compared to that of genistein and estradiol [55] may explain the absence of the preferential mediation of a specific ER subtype. Another point of consideration is that ERα and ERβ may act simultaneously and thus counteract the function of the other receptor subtype [59], [60]. Both genistein and daidzein were shown to activate binding to ERβ at nanomolar concentrations (30 nM and 350 nM, respectively), which are easily achievable levels in humans consuming soy products or supplements. At the concentrations used in these experiments, it is plausible that both receptor subtypes were activated and as such ER subtype specific effects may be diminished.

Caspase 3 has a pivotal role in the apoptotic process. Multiple pro-apoptotic pathways converge on caspase 3 activation in the cell death cascade. Caspase 3 activation may occur through caspase 9 from the mitochondria or from death receptor signaling via caspase 8 as well as through other pro-apoptotic pathways [61]–[63]. More upstream in the apoptotic cascade, the upregulation of Bax is associated with mitochondrial membrane permeabilization and release of pro-apoptotic factors from mitochondria, leading to caspase activation. The ERβ specific effects on Bax may be related to the recent discovery of ERβ localization in mitochondria [64], suggesting a direct estrogenic effect on mitochondria function via ERβ activation and signaling. The ER-mediated reduction of caspase 3 activity and Bax expression by estradiol and isoflavones suggested that these compounds disrupt apoptotic signaling by downregulating key pro-apoptotic factors in the cell death cascade. As multiple apoptotic pathways converge on mitochondria functioning and caspase 3 activation, Bax and caspase 3 represent potential upstream and downstream receptor-sensitive check points for estrogenic compounds to disrupt apoptotic processing in response to neurodegenerative insults.

As isoflavones affect the viability of neurons and cognitive function by acting as an estrogenic agonist, they can also utilize differential distribution and regulation of the ER subtypes, ERα and ERβ in the brain. Microarray experiments have shown that ERα and ERβ regulate different genes [65], [66]. Differences in conformation that occur upon ER binding affects the recruitment of coregulatory proteins, and thus produces differential gene regulation in specific cell types. In addition to tissue or region specific localization of ERs, intracellular localization of ERs may contribute to some of their different mechanisms of action. ERβ has been localized to a greater extent at extranuclear sites and in the cytoplasm for trafficking to the plasma membrane [64], [67], [68]. The extranuclear and membrane localization of ERβ enables its interaction with intracellular signaling cascades to integrate rapid signaling events and classical transcriptional mechanisms [69], [70]. Given the timing of treatment in our studies, both genomic and nongenomic molecular actions may be utilized by isoflavones to confer ER-mediated neuroprotection against Tat.

Despite the success of cART on peripheral viral suppression, protected viral reservoirs in the brain may allow continued release and exposure to toxic viral proteins [10]. The inability of anti-retroviral therapy to prevent the development of neurocognitive dysfunction indicates the need for adjunctive therapies to address the neurodegenerative and subsequent neurological disturbances associated with HAND. Findings in the present study demonstrate that soy isoflavones offer a similar protective effect as endogenous estradiol via a selective estrogen receptor -mediated mechanism against HIV-1 Tat-induced cell death. Isoflavones, acting as selective ER agonists targeting the neuroprotective effects associated with estradiol, may represent a safe and viable neuroprotectant along with cART to improve the neurological health of both men and women with HAND.

## Materials and Methods

### Ethics Statement

All of the experimental procedures using animals were performed in accordance with the recommendations in the NIH Guide for the Care and Use of Laboratory Animals. The relevant animal use protocols were approved by the University of South Carolina Animal Care and Use Committee under the auspices of Animal Assurance Number A3049-01.

### Primary Neuronal Cell Culture

Primary cultured cortical neurons were prepared from 18-day-old Sprague-Dawley rat fetuses [50]. Rat cortices were dissected and incubated for 15 min in a solution of 2 mg/mL trypsin in Ca^2+^- and Mg^2+^-free Hanks’ balanced salt solution (HBSS) buffered with 10 mM HEPES (Invitrogen, Carlsbad, CA). The tissue was then exposed for 2 min to soybean trypsin inhibitor (1 mg/mL, in HBSS) and then rinsed 3 times in HBSS. Cells were dissociated by trituration and distributed to poly-L-lysine coated culture plates with wells containing DMEM/F12 medium (Invitrogen) supplemented with 100 mL/L fetal bovine serum (Sigma Chemicals, St. Louis, MO). After a 24-hr period, the DMEM/F12 medium was replaced with 2% v/v B-27 Neurobasal medium supplemented with 2 mM GlutaMAX and 0.5% w/v D-(+) glucose (Invitrogen). Two-thirds of the neurobasal medium was replaced with fresh medium of the same composition once a week. Cultures were used for experiments after 12 days in culture in serum-free medium and were >95% neuronal as observed by anti-MAP-2 immunostaining.

### Tat_1–86_ Exposure and Experimental Treatment of Cultures

Recombinant Tat_1–86_ (LAI/Bru strain of HIV-1 clade B, GenBank accession no. K02013, Diatheva, Fano, Italy) was added to cell culture medium. Groups of cultures in 24-well plates were exposed to 50 nM Tat. Cell cultures were treated with 17β-estradiol (0.1 nM, 2 nM or10 nM, Sigma) or soy isoflavones (0.05 µM, 0.2 µM or1 µM, genistein (GEN) or daidzein (DAI), Indofine Chemical) for 24 hr prior to Tat exposure and remained present in medium throughout experiments. 17β-estradiol was dissolved in sterile water and diluted in D-PBS. Isoflavones were dissolved in DMSO and diluted in D-PBS. To assess neurotoxicity, the cultures were exposed to Tat for 4, 16, or 24 hr before harvesting. After treatment, medium was removed, cells were washed and lysates collected for ELISA experiments. Cells were also treated with the estrogen receptor antagonist ICI 182,780, the ERα specific antagonist, MPP dihydrocloride, or the ERβ specific antagonist PHTPP (100 nM, Tocris Cookson Inc, Ellisville, MO) 1 hr before estradiol or isoflavone treatment to determine if the effects against Tat toxicity were receptor mediated.

### Cell Viability Assay

Neuronal survival was determined using a Live/Dead viability/cytotoxicity kit (Molecular Probes, Eugene, OR) in rat fetal cortical cell cultures prepared in 96-well plates. In accordance with the manufacturer’s protocol, neurons were exposed to cell-permeate calcein AM (2 µM), which is hydrolyzed by intracellular esterases, and to ethidium homodimer-1 (4 µM), which binds to nucleic acids. The cleavage product of calcein AM produces a green fluorescence (F_530 nm_) when exposed to 494-nm light and is used to identify live cells. Bound ethidium homodimer-1 produces a red fluorescence (F_645 nm_) when exposed to 528-nm light, allowing the identification of dead cells. Fluorescence was measured using a Bio-Tek Synergy HT microplate reader (Bio-Tek Instruments, Inc., Winooski, VT). Each individual F_530 nm_ and F_645 nm_ value on a plate was corrected for background fluorescence (readings obtained from cell cultures (wells) that were not exposed to calcein AM and ethidium bromide) by the microplate reader KC4 software package (Bio-Tek Instruments, Inc., Winooski, VT). For each individual cell culture (well) on a plate, ratios between corrected green and red fluorescence (F_530 nm_/F_645 nm_, Live/Dead ratios) were calculated. All individual relative numbers of live and dead cells were expressed in terms of percentages of average maximum Live/Dead ratio determined for the set of non-treated control cell cultures (8–16 wells) from the same plate: (F_530 nm_/F_645 nm_)_well n_/(F_530 nm_/F_645 nm_)_average max_ × 100%.

### Detection of Apoptotic Proteins (ELISA)

Expression of apoptotic signaling proteins in cell lysates was determined by ELISA [16], [50]. Cell lysates were prepared from cultures grown in 24-well plates. At the time of harvesting, medium was removed and cells were washed 3 times with Dulbecco phosphate-buffered saline, D-PBS, (8 mM Na_2_HPO_4_, 1.5 mM KH_2_PO_4_, 0.137 M NaCl and 2.7 mM KCL at pH 7.4) and lysed with CellLytic™- M mammalian cell lysis buffer (Sigma Chemicals) containing protease inhibitors (protease inhibitors cocktail, Sigma Chemicals). All samples in a group (6 culture wells) were pooled together and protein concentration was determined by the BCA method (Pierce, Rockford, Ill.). Each well of Costar 96-well ELISA plates (Corning Inc, PA) was coated overnight at 4°C using 100 µL of 20 mM carbonate coating buffer, pH 9.6. Cortical cell lysate samples were diluted 1∶10 with D-PBS and 20 µg of each sample were added to the plate wells. After overnight incubation at 4°C, plates were rinsed 5 times with PBST (0.05% Tween 20 in PBS, pH 7.4) and blocked with 1% BSA in PBS for 2 hr at room temperature. After blocking, plates were washed again, as described above, and primary anti-Bax, anti-Bcl-2, anti- active Caspase 9 and anti- active Caspase 3 antibodies (all primary antibodies, Abcam, Cambridge, MA) diluted 1∶5000 or 1∶7500 (caspase 3) in 0.1% BSA-PBST were added to each well except for blanks and no-primary antibody control wells. Plates were kept overnight at 4°C. When the incubation with primary antibodies was completed, plates were again washed 5 times with PBST and secondary antibodies [goat anti-rabbit alkaline phosphatase conjugated, Sigma] diluted 1∶2000 in 0.1% BSA-PBST were added to each well, except for blank and no-secondary antibody control wells. After 2 hr of incubation, the secondary antibody solution was removed, plates were washed 5 times with PBST and 100 µL of BluePhos phosphatase substrate mixture (KPL Research, Gaithersburg, MD) was added to the plate wells. After 30 min of incubation, the absorbance at 650 nm was determined using a Bio-Tek Synergy HT microplate reader. Multiple readings were taken within a 1-hr time period.

### Statistical Analysis

Statistical comparisons were made using one-way ANOVA and Tukey’s multiple comparison tests were used to determine specific treatment effects. Significant differences were set at *p*≤0.05. Data represents mean values ± standard error of the mean (SEM).
